# Can we diagnose Van Gogh?

**DOI:** 10.1192/bjb.2025.10192

**Published:** 2026-02

**Authors:** Nimesh Naran, Robert Howard

**Affiliations:** Core Trainee 3 in Psychiatry, North London Foundation Trust, London, UK; Professor of Old Age Psychiatry, University College London, London, UK

I am writing to share findings and reflections from a case presentation I delivered during our NHS Trust academic programme regarding the psychiatric history of Vincent van Gogh, as well as from a subsequent visit to Arles and Saint-Rémy-de-Provence, the towns where he experienced some of his most severe mental health episodes. I hope these reflections offer useful insights for our ongoing discussions about mental illness, creativity and historical psychiatric practice.

Vincent van Gogh (‘Vincent’) is famously known to have suffered with mental illness, but what exactly he was suffering from remains unclear. Suggested diagnoses have ranged from schizophrenia, bipolar disorder, borderline personality disorder, alcohol use and focal epilepsy to more obscure conditions such as lead poisoning, acute intermittent porphyria and sunstroke.^1^

As a brief background, Vincent did not find his direction in life as an artist until he was 27 years old. In these years of his life, he wrote about periods of low mood, which he described as ‘lying in a deep, dark pit, powerless’,^2^ and led a very unhealthy lifestyle. He was known to drink wine and absinthe (which at the time contained 70% alcohol and had hallucinogenic properties) and wrote about periods of decreased sleep when he painted through the night. He wrote long, incoherent letters to his brother during this time, in which he mentioned being fearful of melancholy. His brother had written about how, for years, Vincent had ‘abandoned what they call conventions’ in his style of dress and demeanour, and that ‘everyone who sees him has said ‘It’s a madman’… Models didn’t want to pose for him, he was forbidden to sit and work in the street because of his volatile disposition.’^3^

In 1888, Vincent tried to establish an artists’ community in Arles, where Paul Gauguin joined him. He experienced his first episode of psychotic symptoms at age 35, which appeared to have been precipitated by an argument with Gauguin. What followed was the incident in which Vincent cut off his ear and presented it to a prostitute in the village. A dramatic scene ensued as the prostitute fainted, and the police found the artist at his home the following morning and took him to Arles General Hospital.

Vincent had two brief admissions to a medical ward, during which he was described as delirious with periods of lucidity. He was discharged but soon became paranoid about being poisoned and was considered a risk to the safety of people in the community, who petitioned the mayor, reporting that Vincent was drinking to excess, wandering and incoherent, and had been sexually inappropriate with women.

Recognising the deterioration in his relationship with the townsfolk, Vincent voluntarily admitted himself to the St Paul de Mausole asylum in Provence. At the initial psychiatric assessment, his doctor reported that Vincent was able to calmly describe his symptoms, and his assessment was that he had ‘suffered an attack of acute mania with visual and auditory hallucinations that led him to mutilate himself by cutting off his ear’ and suspected that he ‘is subject to attacks of epilepsy, separated by long intervals’.^4^

During a year-long admission, Vincent ‘was calm for most of the time, [but] had several attacks lasting for between two weeks and a month; during these attacks, [he was] subject to terrifying fears, and on several occasions he has attempted to poison himself, either by swallowing colours that he used for painting, or by ingesting paraffin.’^5^ Vincent explained these episodes by saying that he had ‘fits of anxiety…melancholy…[and] terrible guilt’ which ‘tend to take an absurd religious turn’.^6^ After discharge, Vincent remained well initially, but his mental state deteriorated and he experienced a recurrence of episodic symptoms, describing ‘extreme sadness and loneliness’. On 27 July 1890, he walked into a wheatfield and shot himself in the chest with a pistol.

Recognising the limitations of historical sources – primarily personal letters written in 19th century French medical terminology – I presented this case to an audience during the academic programme. Attendees were asked whether Van Gogh’s symptoms could be explained by a single diagnosis; 15 of 22 participants (68%) concluded it could not. When asked for the most likely diagnosis, responses varied more widely: 52% selected bipolar I disorder, and 35% chose schizoaffective disorder. Differential diagnoses suggested by attendees also included borderline personality disorder (18%), recurrent depressive disorder with psychotic features and alcohol-induced psychotic disorder (each 14%).

Following this presentation, I visited Arles and St Remy-de-Provence, the two idyllic towns which bore witness to the acute phases of Vincent’s illness. Observing the landscape and making a pilgrimage to the asylum is to immerse oneself in his vivid colour palette, which was inspired by the Provencal countryside – the burnt wheatfields, ochre-washed houses, bright blue skies and deep green cypresses.

In the asylum, Vincent’s room and studio has been preserved as a shrine, and the walls are replete with insightful explanations of his life and illness extracted from a publication by the institution’s medical director, Dr Jean-Marc Boulon: *Van Gogh: Vincent van Gogh’s Life, Works and Illnesses*.^7^ The author describes having contributed to numerous conferences on Vincent’s case, from which he states that most professionals debating this issue have considered bipolar disorders ‘associated with aggravated epileptic fits, provoked or associated with various toxins including absinthe, digitalis, carbon monoxide, [and] tobacco’.

A paper by Nolen et al in 2020^1^ reviewed the letters and interviewed art historians to consider all possible diagnoses that had historically been ascribed to Vincent. The authors concluded that Vincent was likely to have suffered from multiple comorbidities: a probable bipolar mood disorder beginning in early adulthood, combined with borderline personality traits. This clinical picture was further complicated by alcohol use disorder and malnutrition, with psychosocial stressors precipitating acute episodes, including the infamous ear-cutting incident. Reasons for hospital admissions included delirium episodes, probably related to alcohol withdrawal, and severe depressive episodes with psychotic features, ultimately culminating in his suicide. The possibility of focal temporal lobe epilepsy remains unresolved owing to lack of neurophysiological data. Although this sounds like a cumbersome conclusion, several interesting discussion points emerged from this publication and the case presentation.

First, the process of diagnosis in psychiatry has not changed substantially since Vincent’s time, and there is considerable overlap and subjectivity in our diagnostic labels. Important differences include advances in treatments for mental illnesses and investigations that can exclude organic disorders, as well as increasing strides towards finding biomarkers for neuropsychiatric disorders.

The experience of preparing this presentation was not unlike that of assessing a new patient and piecing together the information available from clinical records. It had the same gaps in documentation, and, as with many of our patients who have been receiving care from different mental health services, Vincent accrued several different diagnoses along the way. It remains important to carefully consider comorbid mental health, substance-related and organic disorders when exploring differential diagnoses and making comprehensive management plans.

The presentation highlights the relevance of looking to history in re-evaluating current practice and appreciating the progress we have made, as well as the road ahead. In reviewing the medical records and letters through a modern lens, one might expect to feel somehow superior to our predecessors from almost 140 years ago, but I was pleasantly surprised by the compassion and optimism that the doctors and the townsfolk of Arles demonstrated in their attitudes towards Vincent during his illness.

It also explored the interrelation between creativity and mental illness, with one attendee asking a thought-provoking question – would the nature of Vincent’s art or the resonance of his life have been different if he could have been treated using modern-day medicine? Dr Boulon responds to this question in his work by posing an alternative question, ‘how [Vincent], who was suffering so much, could have been relieved with the medication available’. He reminds us that a contemporary psychiatrist should never lose sight of the fundamental objective of relieving the psychic distress of their patients, ‘even if it is for the purposes of protecting the most beautiful work’.^7^
